# Expression of early angiogenesis indicators in mature versus immature teeth

**DOI:** 10.1186/s12903-020-01313-1

**Published:** 2020-11-12

**Authors:** Javier Caviedes-Bucheli, Luis F. Lopez-Moncayo, Hernan Dario Muñoz-Alvear, Francisco Hernandez-Acosta, Melissa Pantoja-Mora, Angie S. Rodriguez–Guerrero, Alexander López–Ordoñez, Luis E. Díaz, Jose Francisco Gomez-Sosa, Hugo R. Munoz

**Affiliations:** 1 Centro de Investigaciones Odontologicas, Pontificia Universidad Javeriana, Bogota, Colombia; 2 Endodontics Department, Universidad Cooperativa de Colombia, Pasto, Colombia; 3 Engineer School, Universidad de La Sabana, Chia, Colombia; 4 Postgraduate Endodontics Department, Universidad Central de Venezuela, Caracas, Venezuela; 5 Endodontics Department, Universidad de San Carlos de Guatemala, Guatemala City, Guatemala

**Keywords:** Angiogenesis, Human dental pulp, HIF-1α, ANG1, ANG2, TIE2

## Abstract

**Background:**

Proper oxygen balance in the dental pulp is essential for cell metabolism. Angiogenesis in the pulp is a constant process during the life of the tooth. Hypoxia indicators in a tissue, such as HIF-1α, as well as vascular destabilization markers, such as ANG2 and its receptor TIE2, are necessary for angiogenesis. Therefore the purpose of this study is to evaluate the expression of HIF-1α, ANG1, ANG2 and TIE2 in dental pulp as early angiogenesis indicators in teeth with complete and incomplete root development.

**Methods:**

Forty human dental pulps were obtained from freshly extracted third molars divided into two groups: incomplete (n = 20) and complete (n = 20) root development. Dental pulps were stored at − 80 °C, defrosted in an ice bath and re-frozen with their respective thaws to disintegrate the tissue. Three sonication cycles were performed until the tissues were homogenized, then thaw were centrifuged and the supernatant was collected for the detection of the markers to be studied. The samples were processed for the ELISA test using the ELISA-sandwich principle. Student t and Mann–Whitney U tests were performed to determine statistically significant differences between groups.

**Results:**

In the complete root development, HIF-1α, ANG1, ANG2 and TIE2 expressions were significantly higher than their expression in the incomplete root development group.

**Conclusions:**

The angiogenic process seems to be a physiological process in the dental pulp. Angiogenic activity is higher in teeth with mature than immature apex teeth.

## Background

Adequate oxygen concentration is necessary for dental pulp homeostasis [1]. Dental pulp cells require a specific oxygen concentration for ATP production by REDOXs reactions for protein synthesis, which is necessary for its metabolic functions that vary over tooth’s life [2, 3]. Occlusal trauma, mastication, orthodontic movements and even ageing process of the teeth increase the metabolic activity of the dental pulp cells which consumes more oxygen leading to transitory hypoxia [4–7].

HIF-1 is a heterodimeric protein, consisting of two subunits HIF-1α and HIF-1β [3, 8]. Hypoxic dental pulp cells express HIF-1α, which activates a cascade of growth factors in order to start the angiogenic process [9]. HIF-1α also has an oxygen-dependent degradation domain, vulnerable to proteasomal hydroxylation under normoxia. HIF-1β is an oxygen-independent constitutively expressed protein [3, 8].

HIF-1 directs migration of endothelial cells towards the hypoxic environment by transcription genes activation of angiogenic factors, such as vascular endothelial growth factor (VEGF), (ANG1), (ANG2), among others which initiate angiogenesis [3, 10].

The angiogenic growth factors acting in a specific order allow angiogenesis. Angiogenesis is a mechanism of formation of a blood vessel from existing ones to regulate oxygen concentration. This process mainly requires these steps: Blood vessel destabilization, vessel hyperpermeability, endothelial cells proliferation and migration, cell to cell contact, tube formation, vessel stabilization, pericytes differentiation and mesenchymal proliferation [11, 12].

VEGFA is the main pro-angiogenic growth factor which is responsible for survival, proliferation, migration and sprouting of the capillary vessel [1]. Endothelial cells activated by HIF-1 express growth factors receptors such as VEGFR2 and NRP1 in their membrane in order to allow VEGFA to bind them and start the angiogenic process [1, 13].

Another pathway to initiate the angiogenic process is via ANG1 and ANG2, which are the primary ligands of Tyrosine kinase with immunoglobulin-like and EGF-like domains-2 (TIE2) [14, 15]. Vascular stabilization depends of TIE2 phosphorylation [16]. During normoxia, ANG1 binds to its receptor TIE2, forming an ANG1/TIE2 complex, which induces an association between pericytes and endothelial cells, producing vasculature stabilization which acts as an inhibitor of angiogenesis. In contrast, during hypoxia ANG2 binds to TIE2, and the ANG2/TIE2 complex inhibits TIE2 phosphorylation even in the presence of ANG1 [1, 16, 17]. ANG2/TIE2 leads to blood vessel destabilization and in the presence of VEGF promotes migration and proliferation of endothelial cells initiating angiogenesis [15, 17].

In teeth with immature apex, dental pulp is not altered by mastication or bruxism because they have not reach occlusion with the antagonist. Dental pulp of immature apex teeth could have an increased cellular activity related to dentine formation which progressively narrows the root canal until complete apex formation. In these teeth, the existing blood vessels in dental pulp are capable of maintaining normoxia with low ranges of angiogenesis [1]. While in teeth with mature apexes, stimuli such as mastication, bruxism and ageing could induce a cellular activity to increase dentine formation. This higher activity promotes the expression of angiogenic growth factors due to imbalance between oxygen supply and its consumption, in order to form new blood vessels [1, 18].

Therefore, the purpose of the present study is to evaluate the expression of HIF-1α, ANG1, ANG2 and TIE2 as early angiogenesis indicators in teeth with complete and incomplete root development.

## Methods

This study was approved by the ethics committee of the Faculty of Dentistry of the Universidad Cooperativa de Colombia in Pasto, Colombia. All patients taking part in the study were required to sign an informed consent form. Human dental pulp was obtained from 40 freshly extracted non-carious and non-restored third molars randomly selected from different healthy patients. Patients under medication, smokers, or pregnant women were excluded from the study. Sample size was calculated with the TAMAMU 1.1® program.

### Specimen preparation

Samples were divided in two groups of 20 teeth. Pulp samples from immature apexes teeth group were taken from patients aged between 16 and 19 years-old, with fully impacted third molars, with an apical orifice diameter greater than 5 mm mesiodistal or buccolingual. Pulp samples from mature apexes teeth group were taken from patients aged between 17 and 29 years-old, with third molars in normal occlusion, without periodontal disease, and an apical orifice diameter not greater than 0.5 mm. Determination of root development was done both radiographically by millimetre periapical radiographs and visually with an endodontic ruler using an operatory microscope under 20X magnification.

The root surface of each tooth was scraped with a blade to remove the attached periodontal ligament that could contaminate the pulp sample. The teeth were then sectioned using a high-speed handpiece with a Zekrya bur (Dentsply Tulsa Dental, OK, USA) irrigated with saline solution. The pulp sample was obtained and processed, according to Gomez-Sosa et al. [1] methods.

The dental pulp tissues were dried and weighed. Then, they were immersed in PBS buffer (phosphate buffer saline, pH 7.4, 0.1 M), frozen at −80 °C, defrosted in an ice bath, and refrozen for two more cycles with their respective thaws so that the tissue would disintegrate. To complete tissue disintegration, three sonication cycles of 15 s at 30 Hz were used with an intermediate 1 min ice bath between cycles. After tissue homogenization, samples were centrifuged at 6000 rpm during 10 min at 4 °C; the supernatant was collected in another tube for protein quantification with a Bicinchoninic acid assay (BCA, Pierce Chemical Company) for measuring the peptides and receptors to be studied.

### ELISA assay

The ELISA assay uses the Sandwich-ELISA principle. The micro ELISA plate provided in this assay had been pre-coated with an antibody specific to human: ANG1 (Angiopoietin 1, Elabscience, E-EL-H5703), ANG2 (Angiopoietin 2, Elabscience, E-EL-H0008), HIF-1α (Hypoxia Inducible Factor 1 Alpha, Elabscience, E-EL-H1277) and ANG-R-Tie2 (Angiopoietin Receptor Tie2, Elabscience, E-EL-H0340). Standards or samples were added to the micro ELISA plate wells and combined with the specific antibody. Then a biotinylated antibody detection specific for peptide or receptor and Avidin-Horseradish Peroxidase (HRP) conjugate was added successively to each microplate well and incubated. Free components were washed away, and the substrate solution I was added to each well. Only those wells that contain peptide or receptor, biotinylated antibody detection and Avidin-HRP conjugate appeared in colour blue. The enzyme–substrate reaction was terminated by the addition of stop solution turning it to a yellow colour. The optical density (OD) is measured spectrophotometrically at a 450 nm wavelength. The OD value is proportional to the concentration of human peptide or receptor. The concentration of peptide or receptor is calculated by comparing OD of the samples with a standard curve. The experiments were run with 3 experimental replicas. Within the samples, points of the curve were run as samples and in some samples the analyte in question was added (as internal standard). The detection limits for ELISA for the different proteins were 1 × 10^–3^ pmol/mg tissue.

### Statistical analysis

For each protein analysed, descriptive statistics were calculated from three experimental replicates to show the variables behaviour in each group of teeth. A Kolmogorov Smirnov test was applied to determine if the results were parametric or not. Student's t-test was performed to determine statistically significant differences between both groups for variables with parametric behaviour and Mann–Whitney U test for variables with non-parametric behaviour.

## Results

Forty samples of dental pulp tissue were analyzed. The Kolmogorov Smirnov test demonstrates that the ANG1 and ANG2 variables adjusted to normality, while the HIF-1α and TIE2 variables showed a non-parametric behavior.

**Fig. 1:**
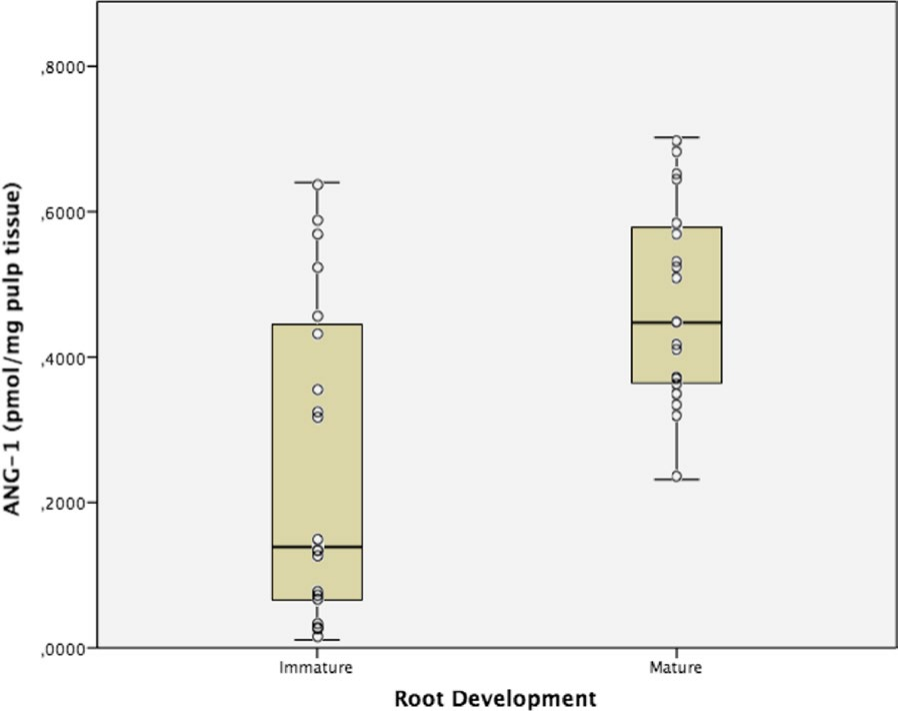
Angiopoietin 1 (ANG-1) expression in human dental pulps from teeth with mature and immature apexes

**Fig. 2:**
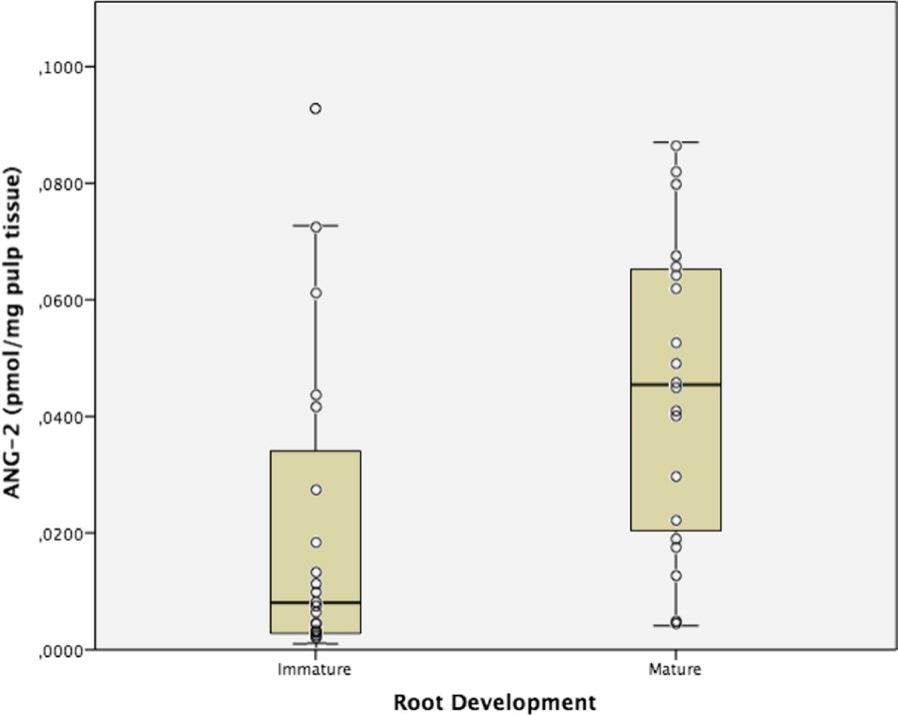
Angiopoietin 2 (ANG-2) expression in human dental pulps from teeth with mature and immature apexes

Figures 1 and 2 show box/dot plot graphs showing data distribution for both angiopoietins. Student’s t test showed statistically significant differences in the expression of both peptides, which were significantly higher in dental pulps of mature apexes teeth (*p* = 0.001 [ANG1] and *p* = 0.008 [ANG2]). The ratio between ANG1:ANG2 was also calculated and found to be 12:1 on immature apex teeth and 11:1 in mature apex teeth.

**Fig. 3:**
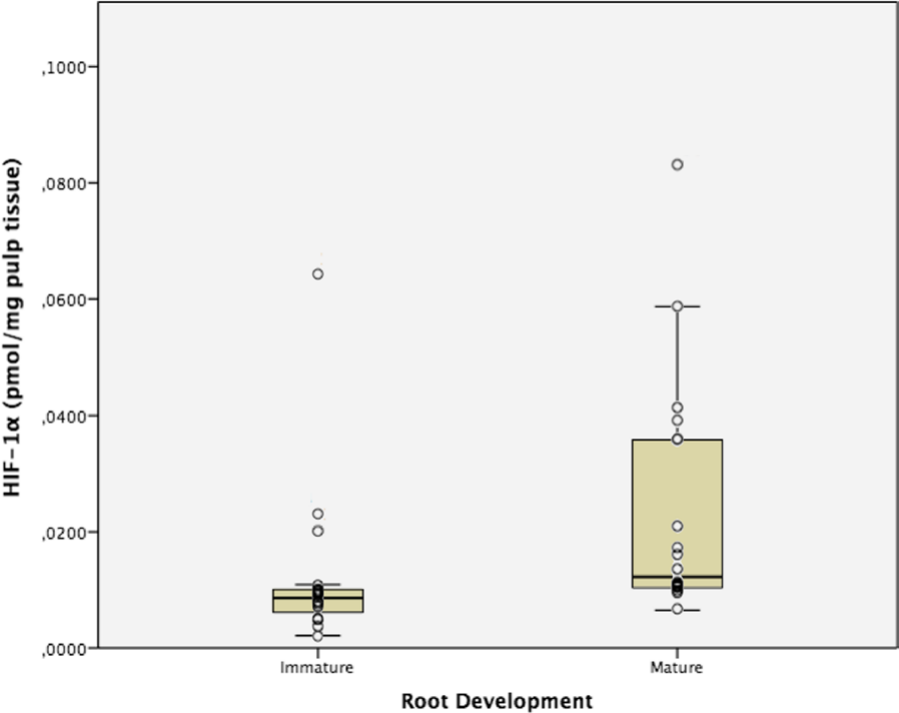
Hypoxia Inducible Factor 1 Alpha (HIF-1α) expression in human dental pulps from teeth with mature and immature apexes

**Fig. 4:**
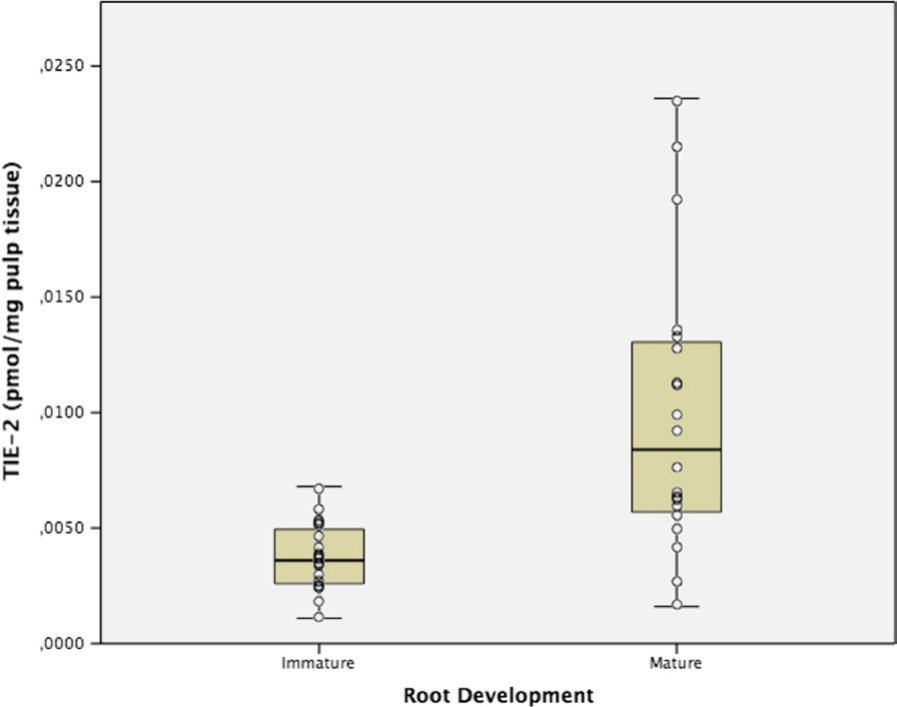
Tyrosine Kinase with Immunoglobulin-like and EGF-like domains-2 (TIE2) expression in human dental pulps from teeth with mature and immature apexes

Figures 3 and 4 show box/dot plot graphs showing data distribution for HIF-1α and TIE2. Mann Whitney’s U test showed statistically significant differences in the expression of both peptides, which were significantly higher in dental pulps of mature apexes teeth (*p* = 0.001 [HIF-1α] and *p* < 0.001 [TIE2]).

## Discussion

One of the pathways that induces angiogenesis as a physiological or defence response in tissues is mediated by HIF-1α and growth factors such as ANG1, ANG2, and its receptor TIE2 [11]. The present study measured the expression of these proteins in dental pulps from immature and mature apexes teeth, which are involved at the initial step of the angiogenesis.

HIF-1α was evaluated, as this protein is oxygen-dependent and its expression indicates that the dental pulp tissue is under hypoxic conditions, because HIF-1α during normoxia is vulnerable to proteasomal degradation [3]. It is well known that for HIF-1 transcription, both activators HIF-1α and β are needed. HIF-1β is a constitutive protein, which remains inactive inside the cell until it binds to HIF-1α in order to transcript HIF-1 to trigger the angiogenic activity [3, 8].

The presence of higher HIF1-α levels in mature apexes teeth indicates lower oxygen concentration in those pulp tissues; and also the possible function of HIF-1 triggering ANG2/TIE2 and VEGFA/VEGFR2 or VEGFA/NRP1 bond in order to start angiogenesis in mature teeth [1]. On the contrary, lower HIF-1α levels in immature apexes teeth could be indicating a lower rate in the angiogenic activity when compares with mature teeth. This suggests two types of cellular activities, proliferation and synthesis. In one hand, cell proliferation, which requires low oxygen concentrations in immature apexes teeth [19] and in the other hand, mineralized tissue formation which requires new blood vessel generation to increase oxygen concentration needed to the metabolic reactions in mature teeth.

A high level of HIF-1α has been observed in teeth under orthodontic movements [20], these findings could be applied to the results of the present research, where stimuli such as dental trauma and mastication in pulps of teeth with mature apexes could raise HIF-1α levels with respect to pulps of teeth with immature apexes that are not under occlusal contact.

It is well known that blood vessel stability is produced via TIE2 phosphorylation by ANG1 [16, 17]. On the other hand, ANG2 has an antagonistic effect over TIE2 inhibiting its phosphorylation which leads to vascular destabilization starting the angiogenic process. Also, it is important to mention that despite of ANG1 is expressed in high levels (11:1 ratio), ANG2 has more affinity to TIE2 and will bind to it [1, 16]. In this research both ligands ANG1 and ANG2 and its receptor TIE2 are significantly higher in dental pulps from teeth with mature apexes than in immature ones, which lead to consider that in mature apexes teeth there are more blood vessels in destabilizing process which suggest a higher angiogenic activity when it compared to the immature apexes teeth.

VEGF is the primary pathway for initiating angiogenesis in dental pulp tissue [13, 21]. However, ANG2 has been studied as a way to initiate the angiogenic response in the dental pulp [1]. In the mentioned study, ANG2 was expressed in both dental pulps from teeth with immature and mature apexes without significant differences. While, in the present study, the expression of ANG2 is significantly increased in mature apex teeth, which could be due to a variation in the inclusion criteria of immature apex teeth group. In the present study, in the immature apex group, teeth with an apical orifice diameter greater than 5 mm were included, while in the study by Gomez-Sosa et al. [1], the group of teeth from the immature apex group consisted of teeth with an apical orifice between 3 and 5 mm. A possible explanation for this difference is that the bigger the apical opening, the less hypoxic dental pulp tissue would be, which supports the findings in HIF-1α in the present investigation. As mentioned before, TIE2 is the receptor of both ligands ANG1 and ANG2 [16], this receptor was found significatively higher in mature apexes teeth than immature ones, which could be due to the increased levels of ANG1 and ANG2 [1] suggesting greater angiogenic activity in matures teeth.

The increased potential for angiogenic activity in mature apexes teeth could be due to intense metabolic activity in order to produce more dentine in teeth with a smaller pulp cavity and denser pulp tissue than immature apexes teeth [1, 12]. This is in accordance with the findings by Gomez-Sosa et al. [1] where an increased potential angiogenic activity was observed in mature apexes teeth associated with all occlusal activities such as mastication, occlusal trauma and bruxism. In this study mature teeth were in occlusal contact while immature ones were not.

Due to a limitation of the present research, in which immunohistochemical assays were not performed, it was not possible to establish which dental pulp cells express each protein. An ELISA test was carried out instead of RT2-PCR because the purpose of the study was to verify the expression of these proteins in the dental pulp, not the genic capacity to synthesize them.

More research is needed to understand the angiogenic process in the dental pulp, its response to different therapeutic alternatives at different root development stages, as well as the different pathways of vascular destabilization and stabilization.

## Conclusion

Within the limitation of the present research, it was concluded that the angiogenic process seems to be a physiological process in the dental pulp. And according to the present findings, the angiogenic activity is higher in teeth with mature apex than in immature apex teeth.

## Data Availability

The datasets used and/or analysed during the current study are available from the corresponding author on reasonable request.
